# A randomised controlled trial of tranexamic acid versus misoprostol in reducing blood loss during caesarean section

**DOI:** 10.4314/gmj.v56i2.1

**Published:** 2022-06

**Authors:** Christian O Ogah, Chidebe C Anikwe, Cyril C Ikeoha, Okechukwu BI Dimejesi, Bartholomew C Okorochukwu, Chidi OU Esike

**Affiliations:** 1 Department of Obstetrics and Gynaecology, Alex Ekwueme Federal University Teaching Hospital Abakaliki, Ebonyi State, Nigeria; 2 Department of Obstetrics and Gynaecology, Federal Medical Centre, Owerri, Imo State, Nigeria

**Keywords:** tranexamic acid, misoprostol, blood loss, caesarean section

## Abstract

**Objective:**

To determine the efficacy of intravenous tranexamic acid versus rectal misoprostol in decreasing intraoperative blood loss during caesarean section (C/S)

**Design and Setting:**

Randomised controlled study involving pregnant women undergoing C/S at Alex Ekwueme Federal University Teaching Hospital, Abakaliki in Nigeria

**Participants and Interventions:**

Five hundred and fourteen women undergoing elective C/S were assigned randomly (257 patients per group) to receive either pre-operative 1000 µg rectal misoprostol or 1000mg intravenous tranexamic acid after spinal anaesthesia. Data from 248 patients were analysed in the misoprostol group, while data from 250 patients were analysed in the tranexamic acid group. Sixteen patients were excluded from analysis; nine had incompletely filled proforma, while seven were lost to follow-up.

**Main outcome:**

Intraoperative blood loss.

**Results:**

The mean intraoperative blood loss was not significantly different between the misoprostol group and the tranexamic acid group (547 ± 183.75ml vs. 551.66 ± 21.74ml, P = 0.157). The mean difference in pack cell volume (PCV) changes was not significant between the groups (2.41±0.95% vs. 2.36±0.56%, P = 0.474). The side effects profile was similar for both groups except for shivering, which was statistically higher among the misoprostol group (RR = 0.70; 95%CI 0.40 - 0.91, P = 0.028).

**Conclusion:**

Intravenous tranexamic acid was comparable to rectal misoprostol in the reduction of blood loss during C/S. Tranexamic acid could act as a good alternative to misoprostol for prophylaxis for blood loss during elective C/S.

**Funding:**

None declared

## Introduction

Every year, over half a million women die of pregnancy-related causes, and about one-quarter of these women die due to primary postpartum haemorrhage (PPH).^1^,^2^ Maternal demise can occur within 2–4 hours of the onset of bleeding^3^, thus making PPH not only the most dangerous obstetric complication and the fastest route to maternal death. Most of these deaths occur in developing regions where maternal health care provision and utilisation are lowest.^2^,^3^ Caesarean section (CS) is a major surgery performed on women worldwide. Its rate is increasing both in developed and in developing countries.^4^–^6^ It is associated with bleeding morbidities and mortality especially in developing countries due to poor access to health care, lack of blood for transfusion and refusal to receive blood.^7^

Patients who undergo caesarean section are at increased risk of developing PPH^8^,^9^, and consequently, about 5% of women require a blood transfusion due to massive blood loss.^10^ This may expose them to the risk of severe anaemia, transfusion reactions, and transmission of blood-borne infections with the attendant sequellae.^11^ Uterine atony is the commonest cause of obstetric haemorrhage during vaginal delivery and caesarean section.^12^ The use of oxytocin in preventing uterine atony has been recommended for every woman in the third stage of labour.^13^ However, despite the routine use of oxytocin as recommended by the WHO, 10–40% of women still need secondary uterotonics such as prostaglandin analogues, which have been used to reduce blood loss during caesarean section.^14^

Misoprostol, in recent times, has been recommended as the first-line drug in preventing excessive postpartum blood loss in many developing countries due to its multiple routes of administration, stability to temperature changes, availability, and cost.^14^ It is a prostaglandin E1 analogue with good uterotonic properties. It has a little adverse effect at therapeutic doses. Misoprostol is as effective as oxytocin in third-stage management without the need for cold chain, parenteral administration and fluid overload associated with oxytocin.^11^ Because of these properties, misoprostol has been evaluated for both prevention of blood loss during caesarean section and treatment of postpartum haemorrhage,^14^ and its effectiveness in reducing blood loss during caesarean section has been established.^11^,^14^ Studies have shown that intra-operative and post-operative blood loss was less in the misoprostol group than in the placebo group. Therefore, they have recommended its use during caesarean section to prevent postpartum haemorrhage.^13^,^15^ However, trauma during caesarean section provokes fibrinolysis, and antifibrinolytics have been suggested to prevent excessive blood loss.^11^,^16^ Antifibrinolytics reduce the incidence of post-operative blood transfusion and its attendant complications. The Clinical Randomization of an Antifibrinolytic in Significant Haemorrhage (CRASH-2) study concluded that tranexamic acid could reduce blood transfusion and mortality in bleeding trauma patients^16^ with no side effects. Also, a randomised controlled trial by Yehia AH and colleagues concluded that tranexamic acid reduces all causes of mortality due to blood loss.^17^ Recently, tranexamic acid was recommended to prevent and treat uncontrollable blood loss following delivery.^18^ Hua et al., in their meta-analysis, concluded that sublingual misoprostol is as effective as intravenous oxytocin for reducing blood loss during caesarean section.^19^ Sahhaf et al.^20^ compared rectal misoprostol and intravenous tranexamic acid in treating PPH for both caesarean section and vaginal delivery. They found that misoprostol was not superior to tranexamic acid in treating PPH.^20^ Despite this fact, tranexamic acid has not been routinely used in obstetric practice to treat or prevent PPH, maybe due to inadequate local research. Therefore, local research is needed to help make recommendations on its use in this regard, especially in developing countries with the highest incidence of PPH. This study aims to establish tranexamic acid's effectiveness in reducing blood loss with subsequent prevention of PPH during the caesarean section.

## Methods

### Study design

The study was an open-label randomised controlled trial on the effectiveness of pre-operative rectal misoprostol and intravenous tranexamic acid in reducing blood loss during caesarean section.

### Study area

The Alex Ekwueme Federal University Teaching Hospital, Abakaliki (AEFUTHA), Ebonyi State, where this study was done, receives referrals from all parts of the state and neighbouring states of Abia, Benue, Cross River, Imo and the Enugu States. The hospital has a very busy obstetrics unit. The antenatal clinics hold Mondays through Fridays, excluding national public holidays, and are conducted by consultants and resident doctors with the help of midwives and other health workers in the Department of Obstetrics and Gynaecology. The total number of antenatal attendees in 2017 was 12,000, with 4,000 deliveries. Ebonyi state has a high maternal mortality rate (MMR) of 1,359 per 100,000 live births, and PPH contributed to 23% of this death.^21^

### Study population

This study was carried out among consenting pregnant women undergoing elective caesarean section at AEFU-THA. They were recruited between April and November 2017.

### Inclusion criteria

The participants were those undergoing elective caesarean section, irrespective of maternal age, with a diagnosis of singleton pregnancy (37 weeks to 42 weeks) and an absolute weight of less than 90 kilogrammes. They must be booked in the hospital. A pregnant woman is assessed to be booked when she has attended at least three antenatal visits, has done the baseline investigations (packed cell volume (PCV), blood group, genotype, venereal disease and research laboratory test (VDRL), hepatitis B virus screening (HbsAg), retroviral screening (HIV), and urinalysis which an obstetrician has evaluated.

### Exclusion criteria

Pregnant women with a history of previous caesarean section scar, asthmatic, multiple pregnancies, antepartum haemorrhage, and anaemic need blood transfusion. Others excluded were pregnant women diagnosed with hypertensive disorders of pregnancy, known allergy to misoprostol or tranexamic acid and a history of bleeding disorder.

The socio-demographic characteristics of the women were obtained from their case notes. A full blood count, blood group, urinalysis, retroviral screening test and cross-matching of two units of blood were routinely done for the women included in the study. Other investigations were done depending on the clinical condition of the women. The pack cell volume was determined 48 hours after surgery. Before surgery, the women were clinically reviewed by the anaesthetist and a paediatrician was always available during surgery for neonatal resuscitation.

### Sample size calculation

The sample size was calculated using the formula for a cross-sectional study.^22^ The prevalence of utilisation of caesarean section of 0.20^23^ was used. A sample size of 257 was calculated after adding a 5 % attrition rate. The sample size of 257 was therefore used for each group giving a total sample size of 514.

### Procedure for randomisation

The participants were randomised utilising computer-generated random numbers using the software Research Randomizer®. Two hundred and fifty-seven were randomly generated from a pool of 514 participants and assigned to the misoprostol group (Group A). The remaining 257 were automatically assigned to the tranexamic acid group (Group B). These numbers were inscribed on a brown envelope and a piece of paper with the inscription misoprostol group or tranexamic acid group was placed inside the respective envelopes and sealed. Participants who met the inclusion criteria and signed the informed consent form were given a sequential study number, and the corresponding numbered opaque sealed envelopes were then allocated to the patient. Randomisation was done by a pharmacist who was not part of the team involved in the study population selection.

### The study interventions

Women randomised to the misoprostol group received 1000µg of pre-operative rectal misoprostol after spinal anaesthesia. Women randomised to the tranexamic acid group received 1000mg intravenous tranexamic acid after spinal anaesthesia. Senior registrars and/or consultants did the surgeries. A Pfannenstiel abdominal incision was used for the caesarean section. A transverse lower segment incision was used to deliver the fetus and the uterus was repaired in two layers using Vicryl 2. The anterior abdominal wall was closed in layers using Vicryl 2 to the rectus sheath, Vicryl 2/0 to the peritoneum, muscle, and subcutaneous layer. The skin was closed subcutaneously using Vicryl 2/0.

### Estimation of blood loss

Blood loss was estimated using an abdominal mop. A uniform 25cm×25cm abdominal mop was used during surgeries. The dry weight of the mop was 55g obtained using a spiral spring scale calibrated in grams (0 — 1200 grams). The abdominal mops were used to dab all the bleeding during the surgery, ensuring that the mops did not soak up amniotic fluid. After the surgeries, the abdominal mops were gathered into a uniform cellophane bag, hung on the scale's hook, and read directly from the calibration to avoid parallax error. The net weight of the mops gotten after subtracting the weight of dry mop from the wet mop gave the blood loss.

The weight of the cellophane was insignificant and was not detected with our scale. Each unit of weight gain in grams is equivalent to 1milliliter of blood loss. 24 The estimation was done for every one of the patients and recorded for the two groups. To serve as a control and to incorporate post-operative haemoglobin estimation, blood loss was estimated from a modification of the Gross formula.^25^ A difference of more than 10mls between formula estimation and abdominal mop estimation of blood loss led to the rejection of the abdominal mop estimation and it is substituted with the value gotten from formula estimation.

Blood loss = BV [Hct (i) - Hct (f)]/ Hct (m)

Where BV was the blood volume calculated from the Body Weight (Blood Volume=Body Weight in kilo-grams x 70 ml) Hct (i), Hct (f) and Hct (m) were the initial, final and mean (of the initial and final) Hematocrits respectively.

### Data analysis

Data were collated, tabulated, and then statistically analysed using Statistical Package for Social Science (IBM SPSS) software (version 22, Chicago II, USA). Continuous variables were presented as mean and standard deviation (Mean ± SD), while categorical variables were presented as numbers and percentages. The Chi-square test (X^2^) was used for comparison between groups for qualitative variables while the student t-test was used for comparison between groups for quantitative variables, the relative risk was used to analyse the effectiveness of each group to prevent excessive blood loss. P-value < 0.05 was considered significant.

### Ethical consideration

Permission to carry out this research was sought and obtained from the Research and Ethics Committee of the Alex Ekwueme Federal University Teaching Hospital Abakaliki. The ethical approval number is FETHA/REC /VOL l /2017/504. The clinical trial was registered with Pan African Clinical Trial Registry. The trial number is PACTR201910644489698. Informed and written consent was obtained from the women before being included in the study.

## Results

Over the study period of eight months, 600 patients were assessed for randomisation into the study. Eighty-six patients did not meet the inclusion criteria. Five hundred and fourteen patients were randomised, 257 patients into each arm of the study. Sixteen patients were excluded from analysis; nine had incompletely filled proforma while seven were lost to follow-up. Data from four hundred and ninety-eight patients were correctly collected, tabulated and analysed.

Data from two hundred and forty-eight patients were analysed in Group A (the misoprostol group), while data from 250 patients were analysed in Group B (the tranexamic acid group). See the Consort flow chart of the patients through the study (Appendix 1)

Table I shows the socio-demographic and obstetric characteristics of participants. In the misoprostol and tranexmic acid groups. There was no statistically significant difference in the socio-demographic variables of the two groups. The majority of the women in both groups were within the 20–34 years of age bracket and were multiparous (Para 2–4). Only 19.4% of the misoprostol group and 25.0% of the tranexamic acid group were primigravid patients while 4.8% of the misoprostol group and 5.6% of the tranexamic acid were grand-multiparous.

**Table 1:** Socio-demographic and obstetric characteristics of participants

Parameters	Misoprostol group (248) (n,%)	Tranexamic acid group (250) (n, %)	Chi- square (X^2^)	p- value
**Age**			0.31	0.577
**<20**	6(2.4)	6(2.4)		
**20–34**	191(77.0)	187(74.8)		
**≥35**	51(20.6)	57(22.8)		
**Educational** **level**			1.76	0.184
**None/primary**	47(19.0)	41(16.4)		
**Secondary**	109(44.0)	101(40.4)		
**Tertiary**	92(37.0)	108(43.2)		
**Marital status**			1.32	0.249
**Married**	230(92.7)	238(95.2)		
**Single**	18(7.3)	12(4.8)		
**Parity**			1.30	0.253
**Primigravida**	48(19.4)	63(25.2)		
**Multi-para**	188(75.8)	173(69.2)		
**Grand multipara**	12(4.8)	14(5.6)		
**Gestational** **age**			10.03	0.001
**Early term**	118(47.6)	100(40.0)		
**Full-term**	79(31.9)	58(23.2)		
**Late-term**	51(20.5)	92(36.8)		

Table 2 showed the blood loss determinants of the groups. There were no statistically significant differences in blood loss determinants such as bedside clothing time, duration of surgery and cadre of the surgeon in both groups. The mean duration of caesarean sections was 47.17±33.55 minutes and 45.13±32.33 minutes for misoprostol and tranexamic acid respectively, this was not statistically significant. The mean duration of caesarean section by consultants and senior registrars was also not statistically different.

**Table 2:** Caesarean section blood loss determinants among the groups

Parameters	Misoprostol Group	Tranexamic acid group	p-value
**Mean bedside clotting time** **(min)**	5.15±1.82	4.90±1.64	0.107
**Mean duration of CS (min)**	47.17±33.55	46.13±32.33	0.724
**Mean duration of CS by consultants** **(min)**	45.00±27.59	43.57±26.57	0.556
**Mean duration of CS by senior** **registrar** **(min)**	49.71±23.30	46.57±22.82	0.129

Table 3 shows a pre-operative pulse rate and blood pres-sure. The mean pre-operative pulse rate, systolic and diastolic blood pressures were within physiological values for the two groups.

**Table 3:** Pre-operative pulse rate and blood pressure

Parameters	Misoprostol group	Tranexamic acid group	RR(95%CI) p-value
**Pre-operative** **systolic BP** **(mmHg)**	132.26±20.96	128.27±15.54	1.01(0.25–4.08), 0.224
**Pre-operative** **diastolic BP** **(mmHg)**	85.44±21.93	81.29±17.18	1.02 (0.25–4.12), 0.224
**Per-operative** **pulse rate** **(beats/minute)**	87.94±15.05	88.82±11.61	0.99(0.24–4.00), 0.703

There was no statistically significant difference in the mean pre and post-operative pulse rate and blood pres-sure between the groups (P > 0.05).

Table 4 shows blood loss estimation during the caesarean section using abdominal mops and formula. The mean number of abdominal mops used per caesarean section was 2.24±0.71 and 2.47±1.01 respectively for misoprostol and tranexamic acid (p > 0.05). The dry and wet weight of the abdominal mobs used during the caesarean section showed a mean weight difference of 547±183.75g for misoprostol and 551.66±21.74g for tranexamic acid (p > 0.05).

**Table 4:** Estimated blood loss and indicators

Parameters	Misoprostol group	Tranexamic acid group	p- value
**Mean difference in the** **weight of the mop (g) (mean** **blood loss from mop --ml)** *	547.05±40.26	551.66±31.99	0.157
**Mean difference in PCV pre** **and post operatively (%)**	2.41±.0.95	2.36±0.56	0.474
**Mean blood loss estimation** **from PCV (ml)**	446.06±140.26	451.66±146.53	0.663
**Blood transfusion (n, %)**	2(0.8)	3(1.2)	0.661
**Additional oxytocic (n, %)**	8(3.2)	9(3.6)	0.818
**Systolic BP <100mmHg (n,** **%)**	8(3.2)	5(2.0)	0.395
**Post-operative pulse rate** **>100beats/minute (n, %)**	14(5.7)	18(7.2)	0.480

g- Gram, *each unit weight gain in grams is equivalent to 1milliliter of blood loss 23

The mean change in pre- and post-operative hemoglobin were 2.40±1.05 and 2.56±0.56 for misoprostol and tranexamic acid respectively (p > 0.05). The mean blood loss estimated from PCV was 446.06±140.26mls and 451.66±146.53mls for misoprostol and tranexamic acid groups respectively. Only two women in the misoprostol group had blood transfusion while it was three women in the tranexamic acid group (RR = 0.67 95%CI 0.11 - 3.98, p= 0.672), additional oxytocic was used for 8 women in misoprostol group vs 9 in the tranexamic group (RR = 0.89 95% CI 0.35 - 2.28, p= 0.818).

Post-operative systolic BP of <100mmHg was recorded for 3.2% and 2% of participants in misoprostol and tranexamic acid respectively (RR = 1.6195% CI 0.53 - 4.86, p = 0.395) while pulse rate greater than 100 beats per minutes at 2 hours post-operatively was found in 5.7 and 7.2% of participants in misoprostol and tranexamic acid group respectively (RR =0.77 95%CI 0.39 - 1.52, p = 0.466).

From Table 5, the mean Apgar scores were not significantly different between the groups. There were no clinical signs of asphyxia between the two groups. Table 6 shows the indications for the caesarean sections; there was no statistically significant difference in the indications for the surgery between the groups.

**Table 5:** Neonatal outcome findings

Neonatal outcome (Apgar score)	Misoprostol group	Tranexamic acid group	p--value
**1^st^ minute**	7.8±1.2	7.6±1.4	0.087
**5^th^ minute**	8.9±0.9	9.0±0.8	0.343

**Table 6:** Indications for Caesarean Section

Indications	Misoprostol group (n,%)	Tranexamic acid group (n, %)	Chi square (p-value)
			1.24 (0.265)
**Breech in primigravida**	12(4.8)	15(6.0)	
**Abnormal presentation**	58(23.4)	59(23.6)	
**Abnormal lie**	78(31.5)	81(32.4)	
**Fetal macrosomia**	39(15.7)	51(20.4)	
**IUGR**	61(24.6)	44(17.6)	
**Total**	248	250	

IUGR – intrauterine growth retardation

**Figure 1:**
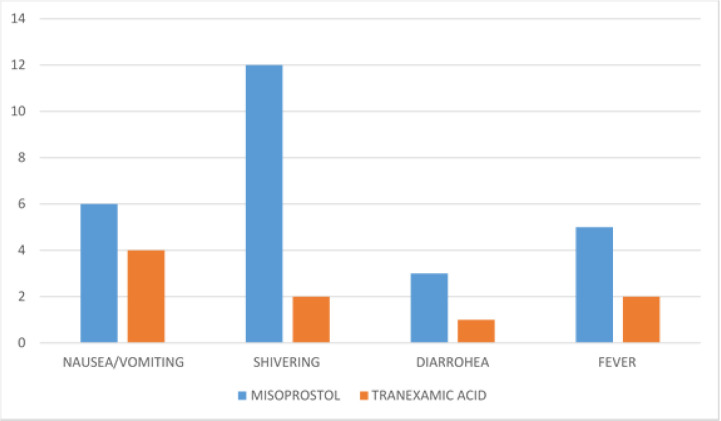
Comparison of the side effect profiles of the groups

As shown in Figure 1, the side effect profiles were similar for both groups except for shivering which was statistically higher among the misoprostol group compared to the tranexamic acid group (RR = 6.04; 95%CI 1.36 -- 26.74, p = 0.017). Both groups had other minor side effects. The risk of diarrhea (RR = 3.02 95%CI 0.31 - 28.87, p = 0.336) nausea/vomiting (RR = 1.51 95%CI0.43 - 5.29, p = 0.517) and fever (RR = 2.52, 95% CI 0.49 - 12.86, p = 0.266) in women that received misoprostol were relatively higher when compared with tranexamic acid group although not significant.

## Discussion

Heavy blood loss during childbirth is the most commonest feared complication of pregnancy and delivery.^26^ This study evaluated the efficacy of intravenous tranexamic acid versus rectal misoprostol in decreasing intra-operative blood loss in women undergoing elective caesarean section in Abakaliki, Nigeria. There were no statistically significant differences in socio-demographic and obstetric characteristics of the patients in the misoprostol and tranexamic acid group in this study.

The blood loss determinants like bedside clotting time, duration of surgery and cadre of the surgeon during caesarean section as shown in Table 2 were comparable in both groups. Confounding variables in the study were thus eliminated by the above findings.

The mean blood loss during caesarean section in the study was 446.06±140.26mls and 451.66±146.53mls for misoprostol and tranexamic acid group respectively which is not statistically different. This suggests that misoprostol and tranexamic acid were equally efficacious in reducing blood loss during childbirth. They might also likely be equal in preventing the development of PPH following CS although not demonstrated in the current study.

Hence, both drugs can serve as a lifesaving alternative when one is not available for the prevention of excessive blood loss in low-risk patients. This finding is similar to the finding of Sahhaf et al.^20^ in Iran and Bose et al. 26 in India, where tranexamic acid was shown to be equally efficacious as misoprostol in reducing blood loss during caesarean section. The mean change in the pre and post-operative packed cell volume in this study was 2.40% and 2.26% for misoprostol and tranexamic acid respectively. This was similar to the work by Bose et al.^26^ where the mean change in pre and post-operative packed cell volume was 3.40% and 3.04% respectively for misoprostol and tranexamic acid using the gravimetric method and also supported by the work of Sahhaf et al. 20 In a related study by Maged et al.^27^ effectiveness of misoprostol in reducing blood loss was demonstrated. In their study, pre-operative use of misoprostol was found to be more effective in reducing intraoperative and post-operative blood loss. This finding was corroborated with our study where intraoperative blood loss was less than 500mls.

The need for blood transfusion was similar in both groups. The proportion of women that were transfused was 0.81% for the misoprostol group and 1.2 % for the tranexamic acid. This is good and encouraging, especially in our environment where blood and blood products are not easily available and any intervention that will assist in reducing the need for blood transfusion is a welcome development. Reduction in the need for blood transfusion following delivery will assist in the prevention of complications of blood transfusion, especially HIV infection which is assuming an epidemic proportion in sub-Saharan Africa.^28^ The use of additional uterotonics to control bleeding was not statistically significant in both groups with only 8 (12%) of the misoprostol group and 9 (13%) of the tranexamic acid needing additional uterotonic. The post-operative vital signs were similar in both the misoprostol and tranexamic acid group in the study. No patient in both groups requires surgical intervention to control blood loss.

This study also showed that the side effect profiles of these two drugs where similar except shivering which was significantly higher in the misoprostol group (RR = 6.04; 95%CI 1.36 - 26.74, p = 0.017). This differs from the study of Bose et al. 26 where the rate of shivering was not statistically different between the misoprostol and tranexamic groups. This difference could be attributed to the differences in study population, design and dose of misoprostol used. In the study of Bose et al., 26 misoprostol was administered sublingually which might explain the increased gastrointestinal symptoms seen among the misoprostol group compared with the Tranexamic group.

Sahhaf et al. 20 on the other hand noted no significant difference in the side effect profile of nausea, vomiting, and diarrhoea among their study population which is in keeping with our findings. They, however, noted a case of thrombo-embolism which was attributed to the effect of pre-eclampsia. In our study, no case of thromboembolism was seen, the difference in the study population might be an explainable reason. During pregnancy, the overall pattern is one of increased coagulation and reduced fibrinolytic capacity which is aimed at preventing PPH following delivery of the placenta, and this could predispose to thrombosis, especially in women with increased odd of it like preeclamptic patients as observed by Sahhaf et al. 20 This then calls for proper patient selection and astute monitoring of the clotting pathway of a patient who is receiving tranexamic acid. On the neonatal outcome, our study shows no significant difference between the two groups and no clinical evidence of neonatal asphyxia. Our finding seems to support the neonatal safety of misoprostol and tranexamic acid although it has been reported that rectal misoprostol use during caesarean delivery might be associated with lower Apgar score.^29^

One of the limitations of this study is because the study was carried out only in our hospital. Because of it, the finding of this study could not be generalised in the area of study; this could be circumvented by conducting further research on this subject involving the recruitment of participants from another mission hospital in the area and even conducting a large randomised controlled study on the place of tranexamic acid vs misoprostol in preventing excessive blood loss following caesarean section. The fact that the study was done on women with low risk for blood loss makes it difficult the extrapolation of the findings to patients with high risk. The hemostatic condition of the patient may have been better accessed with a clotting profile than bedside clotting time as in our study. Our study is a randomised control study which adds to the strength and veracity of our findings. The study population selection in the different groups was based on equal chance. It thus removed the human bias even though our work was a hospital-based study. The relatively large sample size of the total number of women studied contributes to the strength of our findings. Blood estimation was done using an abdominal mop which is prone to error and was supplemented with the incorporation of post-operative haemoglobin estimation using a mathematical formula^25^ that is more accurate. This inclusion of a mathematical formula thus increases the authenticity of blood loss estimation in our study.

## Conclusion

From this study, Tranexamic acid was comparable to misoprostol in the reduction of blood loss during caesarean section, reduction in the need for blood transfusion and the need for additional uterotonics to control blood loss. Therefore, tranexamic acid could act as a good alternative to misoprostol for prophylaxis for blood loss during elective caesarean section. We recommend that tranexamic acid should be added to the essential drug list in our centre for the reduction of blood loss in women undergoing elective caesarean sections. A multi-centre randomised study with a larger sample size is needed especially including women at increased risk of PPH in the above subject to make a generalised recommendation for the general population of Nigeria.
